# Outbreak of Diarrhea Caused by a Novel *Cryptosporidium hominis* Subtype During British Military Training in Kenya

**DOI:** 10.1093/ofid/ofae001

**Published:** 2024-01-03

**Authors:** Romeo Toriro, Scott Pallett, Stephen Woolley, Charlie Bennett, Isra Hale, Jennifer Heylings, Daniel Wilkins, Thomas Connelly, Kennedy Muia, Patrick Avery, Andrew Stuart, Laura Morgan, Mark Davies, William Nevin, Oliver Quantick, Guy Robinson, Kristin Elwin, Rachel Chalmers, Daniel Burns, Nicholas Beeching, Thomas Fletcher, Matthew O’Shea

**Affiliations:** Army Medical Services, Robertson House, Royal Military Academy Sandhurst, Camberley, Surrey, UK; Department of Clinical Sciences, Liverpool School of Tropical Medicine, Liverpool, Merseyside, UK; Centre of Defence Pathology, Royal Centre for Defence Medicine, Queen Elizabeth Hospital Birmingham, Birmingham, UK; Department of Clinical Sciences, Liverpool School of Tropical Medicine, Liverpool, Merseyside, UK; Centre of Defence Pathology, Royal Centre for Defence Medicine, Queen Elizabeth Hospital Birmingham, Birmingham, UK; Centre of Defence Pathology, Royal Centre for Defence Medicine, Queen Elizabeth Hospital Birmingham, Birmingham, UK; 3 Medical Regiment, Fulwood Barracks, Preston, Lancashire, UK; 28 (C-CBRN) Engineer Regiment, Rock Barracks, Woodbridge, Suffolk, UK; 2nd Battalion the Rifles, Thiepval Barracks, Lisburn, UK; 29 Public Health Division Medical Group, HQ 3 (UK) Division, Bulford, Wiltshire, UK; British Army Training Unit (Kenya), Nanyuki, Kenya; Defence Primary Healthcare, Medical Centre, Nanyuki, Kenya; Defence Primary Healthcare, Medical Centre, Nanyuki, Kenya; HQ 1st (UK) Division, Imphal Barracks, York, Yorkshire, UK; British Army Training Unit (Kenya), Nanyuki, Kenya; Department of Clinical Sciences, Liverpool School of Tropical Medicine, Liverpool, Merseyside, UK; Army Health, Army Headquarters, Andover, Hampshire, UK; *Cryptosporidium* Reference Unit, Public Health Wales Microbiology, Singleton Hospital, Sketty, Swansea, Wales, UK; Swansea University Medical School, Swansea, Wales, UK; *Cryptosporidium* Reference Unit, Public Health Wales Microbiology, Singleton Hospital, Sketty, Swansea, Wales, UK; *Cryptosporidium* Reference Unit, Public Health Wales Microbiology, Singleton Hospital, Sketty, Swansea, Wales, UK; Swansea University Medical School, Swansea, Wales, UK; Royal Centre for Defence Medicine, Birmingham, UK; Department of Clinical Sciences, Liverpool School of Tropical Medicine, Liverpool, Merseyside, UK; Department of Clinical Sciences, Liverpool School of Tropical Medicine, Liverpool, Merseyside, UK; Royal Centre for Defence Medicine, Birmingham, UK; Centre of Defence Pathology, Royal Centre for Defence Medicine, Queen Elizabeth Hospital Birmingham, Birmingham, UK; Institute of Immunology and Immunotherapy, College of Medical & Dental Sciences, University of Birmingham, Birmingham, UK

**Keywords:** *Cryptosporidium*, diarrhea, DNA extraction and speciation, military population, outbreak

## Abstract

**Background:**

We report clinical, epidemiological, and laboratory features of a large diarrhea outbreak caused by a novel *Cryptosporidium hominis* subtype during British military training in Kenya between February and April 2022.

**Methods:**

Data were collated from diarrhea cases, and fecal samples were analyzed on site using the multiplex polymerase chain reaction (PCR) BioFire FilmArray. Water was tested using Colilert kits (IDEXX, UK). DNA was extracted from feces for molecular characterization of *Cryptosporidium A135*, *Lib13*, *ssu rRNA*, and *gp60* genes.

**Results:**

One hundred seventy-two of 1200 (14.3%) personnel at risk developed diarrhea over 69 days. One hundred six primary fecal samples were tested, and 63/106 (59.4%; 95% CI, 0.49%–0.69%) were positive for *Cryptosporidium* spp. Thirty-eight had *Cryptosporidium* spp. alone, and 25 had *Cryptosporidium* spp. with ≥1 other pathogen. A further 27/106 (25.5%; 95% CI, 0.18%–0.35%) had non-*Cryptosporidium* pathogens only, and 16/106 (15.1%; 95% CI, 0.09%–0.23%) were negative. *C. hominis* was detected in 58/63 (92.1%) *Cryptosporidium* spp.–positive primary samples, but the others were not genotypable. Twenty-seven *C. hominis* specimens were subtypable; 1 was *gp60* subtype IeA11G3T3, and 26 were an unusual subtype, ImA13G1 (GenBank accession OP699729), supporting epidemiological evidence suggesting a point source outbreak from contaminated swimming water. Diarrhea persisted for a mean (SD) of 7.6 (4.6) days in *Cryptosporidium* spp. cases compared with 2.3 (0.9) days in non-*Cryptosporidium* cases (*P* = .001).

**Conclusions:**

Real-time multiplex PCR fecal testing was vital in managing this large cryptosporidiosis outbreak. The etiology of a rare *C. hominis gp60* subtype emphasizes the need for more genotypic surveillance to identify widening host and geographic ranges of novel *C. hominis* subtypes.

Cryptosporidiosis typically involves small bowel infection, resulting in watery diarrhea that may be accompanied by abdominal cramps, vomiting, and low-grade fever [1, 2]. Infection usually follows fecal–oral transmission or consumption of food or water contaminated with protozoan parasite *Cryptosporidium* oocysts. Fewer than 10 oocysts can cause infection [3]. Symptoms typically self-resolve within 2 weeks in the immunocompetent, although there is risk of chronic and/or severe diarrhea in immunosuppressed populations [1, 2]. Nucleic acid amplification testing (NAAT) for *Cryptosporidium* spp. replaced traditional light microscopy ∼10 years ago in some industrialized countries and is now included in many commercial multiplex fecal pathogen panel tests, although sensitivity for unusual species may be affected by target gene choices [1, 4, 5]. However, light microscopy remains the standard routine diagnostic method in many countries. Environmental water testing requires high-volume sample filtration and specialist laboratories, making pathogen identification challenging in low-resource settings. Point-of-care multiplex polymerase chain reaction (PCR) for diarrhea diagnostics has been found to be robust and effective for diagnosis of diarrhea in these settings [6], but molecular methods used for species identification and subtyping, including PCR sequencing, are rarely available in such environments.

Cryptosporidiosis has previously been described in military personnel from the United States (El Salvador) [7], Germany (Germany) [8], and France (French Guiana/France) [9, 10], either in small numbers among other infectious causes of gastroenteritis [7] or in outbreak settings through epidemiological and serological identification of likely cases [8, 9]. While there are no such reports of acute cryptosporidiosis in military populations in Sub-Saharan Africa, it remains a potential medical problem. Factors exacerbating risk can include limited point-of-care diagnostics, clinical management options, infection prevention/control measures, and effective water treatment technologies [11]. *Cryptosporidium* oocysts have been identified in African bodies of water, wastewater, treated effluents, and drinking water [12], highlighting the potential for transmission of endemic waterborne *Cryptosporidium* to local and traveler populations [13]. *Cryptosporidium* spp. are highly resistant to chlorination [1, 2, 14], rendering standard infection control measures less effective. We describe a cryptosporidiosis outbreak investigation supported by contemporaneous molecular diagnostics among British military personnel training in a travelers’ diarrhea risk-prone area of rural Kenya [15].

## METHODS

### Timeline of Investigation

British Army personnel arrived in Nanyuki, Kenya, in early February 2022. Following increasing reports of profuse watery diarrhea among 1200 personnel between February 11 and 13, an outbreak investigation was declared on February 15, 2022, and included early environmental as well as epidemiological investigations. On February 21, multiplex PCR became available, and on-site testing of fecal samples from probable cases commenced. PCR-positive fecal samples and water samples were repatriated to the *Cryptosporidium* Reference Unit (CRU; Swansea, UK) for molecular characterization of the outbreak [13, 14].

### Outbreak Investigation

A multidisciplinary investigation team was convened; outbreak measures were formulated and agreed upon, including case definitions, testing of fecal samples from probable cases, environmental investigation of possible sources, and control measures. All personnel reporting sick with diarrhea were eligible for study inclusion. Probable cases were defined as individuals on-site and in any of the training area locations experiencing new onset of ≥2 profuse watery/semisolid stools per day, accompanied by ≥1 of the following: urgency, temperature ≥37.8°C, abdominal pain/cramps, nausea, vomiting, loss of appetite, or close contact with another case [15–17]. Confirmed cases were defined as meeting the probable case definition accompanied by a contemporaneous positive fecal PCR result. Diarrhea was considered to have ceased if 48 hours had passed since the last unformed stool. Duration of diarrhea was measured in days from first presentation to time to last unformed stool [18].

### Epidemiological Investigations

Epidemiological investigations were conducted to establish possible infection sources and included analysis of individual activity timetables, sleeping arrangements, and assigned ablution locations to investigate any links between any of these with the previous visit, or close contact with anyone who had visited the suspected point source. Lack of capacity to conduct standardized interviews of cases and controls precluded initiation of a formal case–control study to implicate likely sources of infection. Cases were isolated, managed as clinically indicated [16, 19], and outbreak data were collated. The investigation continued PCR testing of all presenting cases until April 20, 2022.

### Public Health Interventions

We adopted the following public health measures: (i) restricted access to adventure training sites as a short-term measure. Long-term measures included (ii) removing asymptomatic military food handlers from culinary duties until they provided a negative fecal PCR sample, (ii) isolation of symptomatic cases, and (iii) increased handwashing/handwashing facilities.

### Multiplex PCR Testing of Fecal Samples on Presentation

Fecal samples from probable cases were collected and tested immediately (or within 48 hours from remote locations). Individuals self-collected fecal samples, which were divided for immediate PCR, and the remainder were kept in cold storage or ambient temperature–suitable media for future analysis. On site, fresh fecal samples were analyzed for 22 pathogen targets (Supplementary Table 1) using the multiplex PCR BioFire FilmArray gastrointestinal (GI) panel, in accordance with the manufacturer's guidelines (bioMérieux, Marcy-l'Étoile, France) [6, 20]. We hypothesized that it was highly probable for asymptomatic personnel to be negative for *Cryptosporidium* spp. within ≤5 days of arrival to Kenya, and fecal samples were collected from 15 of these individuals for testing as negative quality controls.

### Environmental Investigation of Infection Source

We examined the link between cases, probable cases, and contacts through continuing epidemiological investigations, water testing, and fecal PCR results to determine a hypothesis for transmission. We identified risk sources such as potable drinking and ablution water, water bowsers supplying drinking water to various remote training locations, treated recreational swimming pools, and freshwater leisure activity adventure training sites, all of which are accessible to paying guests and off-duty service personnel. From these sources, we collected water samples from 8 water taps, 7 commercial adventure training sites, and local recreational swimming pools. We then quantified these samples for total fecal coliforms and *Escherichia coli* using Colilert water testing kits (IDEXX, Newmarket, UK). We also conducted off-label PCR analysis of the water samples for *Cryptosporidium* spp. High-volume sampling recommended for detection of *Cryptosporidium* spp. was not possible on site; therefore only six 150-mL water containers were filled then repatriated to the United Kingdom for further analysis at the CRU.

### DNA Extraction and Speciation

Repatriated water samples were centrifuged at 1550*×g* for 10 minutes. DNA was extracted directly from stools and pellets from water samples using a spin-column kit (QIAamp Fast DNA Stool kit, Qiagen) and tested for *C. hominis* and *C. parvum* using a duplex real-time PCR targeting the *A135* and *Lib13* genes, respectively [21]. If DNA was not detected, samples were retested by real-time PCR targeting the *ssu rRNA* gene; amplicons were analyzed by Sanger sequencing [22].

DNA samples from stools underwent amplification of a ∼300-bp fragment of the *gp60* gene using real-time PCR combined with high-resolution melting (HRM) analysis, followed by one-direction Sanger sequencing of amplicons. The real-time PCR assay used a previously described CRU primer cocktail [23] with a 700-nM final concentration of forward and reverse primers used in an HRM master mix (Type-it HRM PCR kit, Qiagen) at 1× final concentration. The assay was run on the Rotor-Gene Q 5-plex instrument (Qiagen) as follows: hot start at 95°C for 5 minutes, followed by 95°C for 10 seconds, 61°C for 30 seconds, and 72°C for 15 seconds for a total of 45 cycles. The HRM was run from 78°C to 86°C, and any samples displaying amplification and a typical melting curve were sequenced in the reverse direction only (Source Bioscience).

Sequence traces were analyzed using Chromosol (Technelysium Pty Ltd.), and subtypes were identified from microsatellite repeat units and downstream regions using previously described nomenclature [24, and Lihua Xiao, personal communication, 2023]. PCR-negative DNA samples for any of the loci were desiccated (Concentrator Plus, Eppendorf) and PCRs repeated. For submission of a *gp60* sequence to GenBank, a longer PCR product was produced by nested PCR (∼800 to 850 bp) [25] and sequenced in both directions.

To infer relationships between *gp60* subtype families, a phylogenetic tree was constructed using Mega 5 [26] using the newly generated sequences and representative sequences of different *C. hominis* and *C. parvum* subtypes obtained from NCBI's GenBank. In this approach, sequences were trimmed to remove the hypervariable microsatellite region, which could introduce more variation than the differences between families, then aligned and analyzed.

### Clinical Management

When clinically indicated, patients were admitted to the medical center for supportive management and/or isolation. Treatment with nitazoxanide was offered to cryptosporidiosis cases if diarrhea was incapacitating and/or completely prevented planned activities, or persisted for ≥7 days [1–3, 16, 27]. Azithromycin was offered to those with severe symptoms related to bacterial pathogens [16, 17, 19].

### Statistical Methods and Ethics

Clinical data from remote locations were collated on bespoke military forms for entry into encrypted electronic patient records stored securely in the medical center. Cases were linked to individual investigation results via unique identifiers, then anonymized before further analysis. Data were entered into Microsoft Excel and cleaned before descriptive tabulations and univariate comparison of variables using analysis of variance or the chi-square test as appropriate. Key outcomes included stool frequency, duration of diarrhea (defined as “day of presentation to time to last unformed stool” [18, 19]), peak recorded temperature, and time in isolation. No consent is required for routine clinical and outbreak management data collation. However, prior Ministry of Defence Research Ethics Committee approval (2076/MODREC/21) was granted in 2021, with an amendment agreed upon in 2022 to enroll permanent staff based in Kenya meeting the eligibility criteria.

## RESULTS

### Outbreak Epidemiology

**Figure 1.**
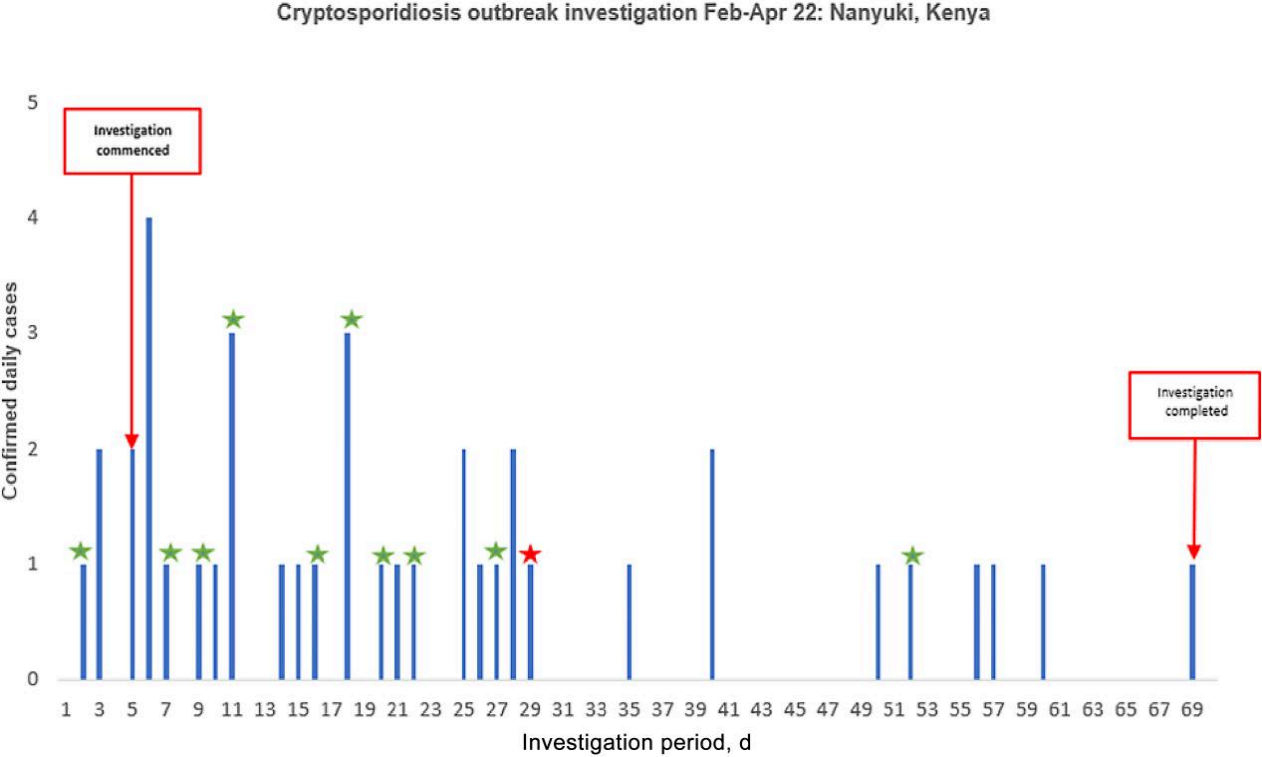
Epidemic curve showing distribution of positive fecal PCR results among confirmed *Cryptosporidium* spp. cases in a British military population, Kenya, February–March 2022. Timing and distribution of PCR-positive *Cryptosporidium* spp. cases. An outbreak was declared on February 15, 2022 (day 5), and fecal testing continued until April 20, 2022 (day 71). *Cryptosporidium* cases which were later confirmed as ImA13G1 subtype presented between days 2 and 52. A single case of *Cryptosporidium* which was confirmed as leA11G3T3 subtype is also shown, and presented on day 29. Abbreviation: PCR, polymerase chain reaction.

Between February 11, and April 20, 2022, 172/1200 (14.3%) individuals presented with symptoms meeting the probable case definition. One hundred six of 172 (61.6%) individuals (85.6% male; median [IQR] age, 24.5 [22–28.8] years) provided fecal samples for testing (Figure 1). Absence of suitable storage facilities precluded sampling in 51/172 (29.7%) individuals presenting before availability of FilmArray capability. There were logistical challenges in retrieving samples for the remaining 15/172 (8.7%) individuals. One asymptomatic control sample was analyzed for every 16 clinical samples on each of 2 FilmArrays, and all tested negative.

One hundred twenty-four patient samples were tested by FilmArray, comprising 106 primary samples from probable cases and 18 repeat samples, including from 3 food handlers. Ninety of 106 (84.9%) primary samples tested positive, and 16/106 (15.1%; 95% CI, 0.09%–0.23%) were negative for all targets. Of the primary samples, 63/106 (59.4%; 95% CI, 0.49%–0.69%) tested positive for *Cryptosporidium* spp. Further breakdown according to etiological subgroupings was as follows: *Cryptosporidium* spp. only: 38/106 (35.8%; 95% CI, 0.27%–0.46%); *Cryptosporidium* spp. and other pathogens: 25/106 (23.6%; 95% CI, 0.16%–0.33%); and non-*Cryptosporidium* spp.: 27/106 (25.5%; 95% CI, 0.18%–0.35%) (Table 1). Five *Cryptosporidium* PCR–positive samples underwent microscopy at a local laboratory and were all positive, independently confirming on-site FilmArray results. Eleven of 18 cases remained positive for *Cryptosporidium* spp. on repeat testing.

**Table 1.** Positive PCR BioFire FilmArray PCR Results in 90/106 (84.9%) Confirmed Cases of Infectious Diarrhea in a British Military Population, Kenya, February–March 2022

Group 1 *Cryptosporidium* spp. Only	No.	Group 2 Mixed *Cryptosporidium* spp. With Other Pathogens	No.	Group 3 Non-*Cryptosporidium* spp. Only	No.
	38		25		27
		EPEC	9	EAEC, EPEC	4
		ETEC	4	*Campylobacter* spp.	3
		EAEC, EPEC	3	EPEC	3
		astrovirus	2	EAEC, ETEC, EPEC	2
		*Campylobacter* spp.	2	EAEC, ETEC, EPEC, sapovirus or rotavirus	2
		EAEC, ETEC, EPEC	1	EPEC, ETEC	2
		EAEC, EPEC, astrovirus	1	STEC	2
		EPEC, *Clostridiodes difficile*	1	*Campylobacter* spp., EPEC	1
		EPEC, ETEC	1	EAEC	1
		*Salmonella* spp.	1	EAEC, STEC	1
				EAEC, STEC, ETEC	1
				EAEC, STEC, ETEC, rotavirus	1
				EPEC, STEC	1
				ETEC, STEC	1
				*Entamoeba histolytica*	1
				*Giardia* spp.	1

Abbreviations: EAEC, enteroaggregative *Escherichia coli*; EPEC, enteropathogenic *E. coli*; ETEC, enterotoxigenic *E. coli*; PCR, polymerase chain reaction; STEC, Shiga-like toxin–producing *E. coli*.

In the first 4 weeks of the investigation, the majority of stools from personnel with diarrhea contained *Cryptosporidium* spp., with or without other pathogens. As case numbers declined in later weeks, a wide variety of bacterial, viral, and other pathogens were detected, and *Cryptosporidium* spp. were in the minority (Table 2).

**Table 2.** Pathogen Distribution Profile Over 12 Weeks Demonstrating Change From an Initial *Cryptosporidium* spp.–Predominant Outbreak in Weeks 1–4 to a Mixture of Pathogens With *Cryptosporidium* spp. in a Minority of Cases in Weeks 7–8; Other Pathogen Distributions Are as Listed in Table 1

Week	No.	Group 1 *Cryptosporidium* spp. Only, No. (%)	Group 2 *Cryptosporidium* spp. and Other Pathogens, No. (%)	Group 3 Other Pathogens Only, No. (%)	No Pathogens, No. (%)	Repeat Samples
1–2	5	5 (100)	0	0	0	0
3–4^a^	36	16 (44)	14 (39)	3 (8)	3 (8)	7
5–6	36	11 (31)	8 (22)	7 (19)	10 (28)	7
7–12	29	6 (21)	3 (10)	16 (55)	4 (14)	4
Total	106	38 (36)	25 (24)	26 (25)	17 (16)	18

PCR BioFire FilmArray test results from February 21, 2023. Five samples collected before FilmArray capability were tested on February 21, 2022, alongside other freshly collected samples. Thirty of 36 (83%) of all samples tested in weeks 3–4 contained *Cryptosporidium* spp. compared with 9/29 (31%) in weeks 7–8.

Abbreviation: PCR, polymerase chain reaction.

^a^February 21, 2022—beginning of week 3 when all PCR testing began.

### Clinical Management of Patients

Clinical data were collated for 51/90 (56.7%) diarrhea cases with positive first FilmArray results. There was no significant difference between the *Cryptosporidium* spp. only and *Cryptosporidium* spp. and other pathogens groups: duration of diarrhea, number of stools passed per day, peak recorded temperature, associated vomiting or abdominal cramps, or time in isolation (Supplementary Table 2). The mean (SD) duration of diarrhea was greater in the *Cryptosporidium* spp. only and *Cryptosporidium* spp. and other pathogens groups combined, at 7.6 (4.6) days compared with 2.3 (0.9) days for non-*Cryptosporidium* spp. (*P* = .001), as was time in isolation at 8.2 (4.6) vs 3.3 (1.1) days, respectively (*P* = .002). There were no other significant differences between these groups. Azithromycin and nitazoxanide were each offered to 7 patients with more severe/prolonged symptoms. Numbers were insufficient for statistical comparison of effectiveness.

### Molecular Characterization (*A135*, *Lib13*, *ssu rRNA*, *& gp60* Genes) and Phylogenetic Analysis

**Figure 2.**
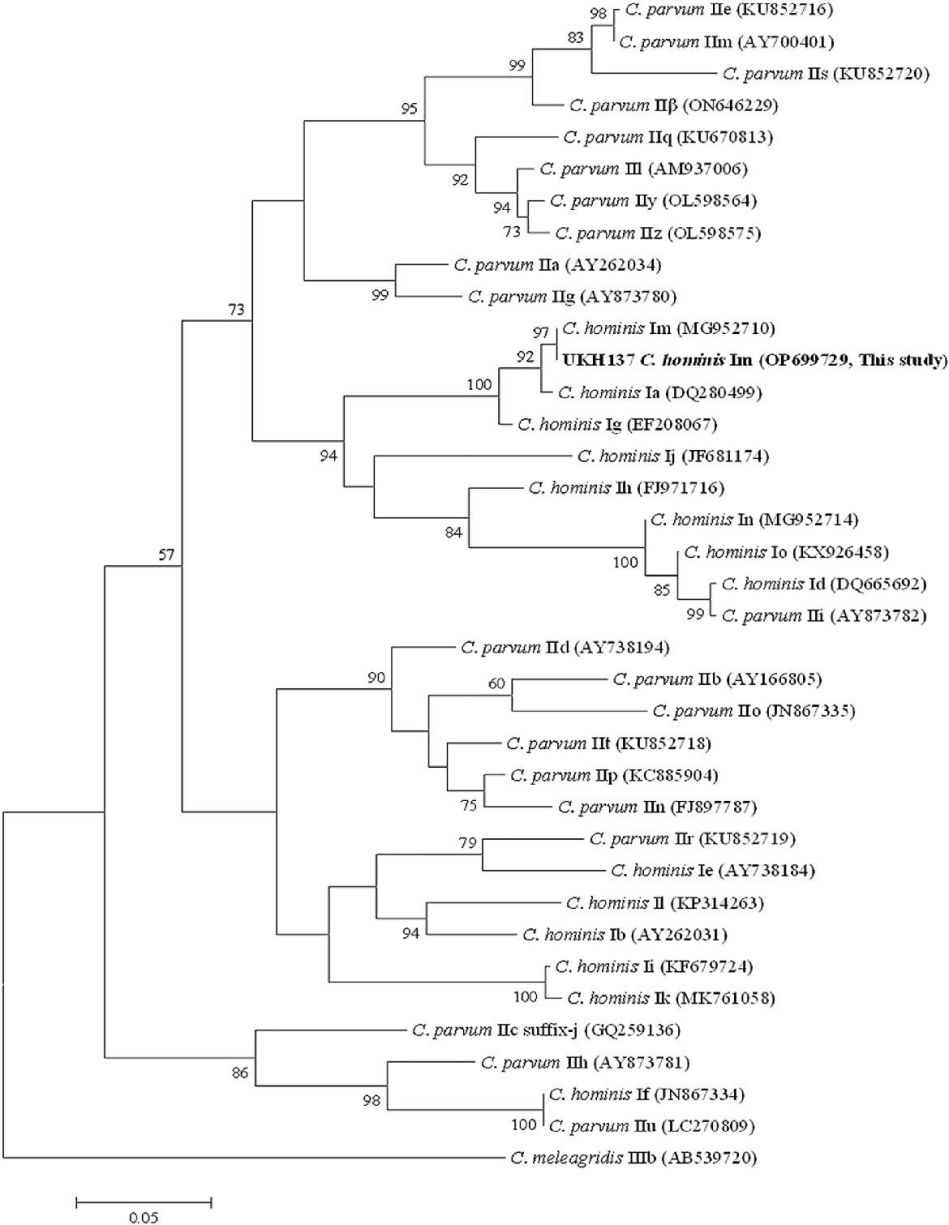
Phylogenetic relationship between *Cryptosporidium* subtype families. Phylogenetic relationship *C. hominis* and *C. parvum* subtype families as inferred by a maximum likelihood analysis of partial nucleotide sequences (623–774 bp depending on subtype family) from the 60 Kda glycoprotein (*gp60*) gene. Bootstrap values >50% from 1000 pseudo-families from the 60 Kda glycoprotein (*gp60*) gene. Bootstrap values >50% from 1000 pseudo-replicates are shown on nodes. GenBank accession numbers for representative *gp60* families are shown in brackets, and the sequence generated in this study is from a representative isolate (UKH137).

Sixty-two cases provided 74 samples (including 12 repeats from 7 individuals) positive for *Cryptosporidium* spp. by FilmArray. Samples underwent molecular characterization of the *A135*, *Lib13*, *ssu rRNA*, and *gp60* genes. Fifty-eight of 74 (78.4%) samples could be genotyped, and all were *C. hominis*. Twenty-six case specimens of the unusual *gp60* subtype ImA13G1 were identified, suggesting a common infection source, and 1 representative specimen from these was placed on GenBank (accession number OP699729). A different subtype (IeA11G3T3) was identified in the fourth sample from a food handler. The remaining 47/74 (63.5%; including 3 from the food handler) samples did not amplify in the *gp60* PCR. The phylogenetic relationship between *C. hominis* and *C. parvum* subtype families is shown in Figure 2.

### Summary of Patient Interviews and Investigation of Source of Infection

One hundred fifty-four of 172 (89.5%) patient interviews were conducted. Molecular characterization of samples from the initial case (Patient 2) showed an identical sequence of ImA13G1 isolated in a further 25 samples from those developing diarrhea later in the outbreak. Of these cases, clusters of the identical subtype were seen among those sharing sleeping quarters (10 individuals) or ablutions (7 individuals). A *gp60* subtype, IeA11G3T3, was detected in a single specimen linked to a food handler presenting with profuse watery diarrhea for ≥7 days and similar exposure history to other cases. Diarrheagenic *E. coli* (4 ETEC, 2 EPEC, 1 EPEC with EAEC, and 1 EAEC with EPEC and ETEC) was found among cases who had eaten at local restaurants before symptom onset, while a cluster of cases associated with *Campylobacter* spp. was related to poultry consumption during a survival exercise.

### Environmental Testing

Fifteen separate water exposure sites were tested, including 7 recreational sites and 8 water bowsers in the training area. All freshwater activity recreational sites were found to have evidence of fecal coliforms. Water tests from the suspected point source recorded >2419.6 fecal coliforms cfu/100 mL and 98 *E. coli* cfu/100 mL, while all water bowsers tested negative (Supplementary Table 3). *Cryptosporidium* spp. DNA was not detected by on-site PCR FilmArray analysis or by later PCR analysis of the repatriated water samples at the CRU targeting the *A135* and *Lib13* and *ssu rRNA* genes.

## DISCUSSION

We describe the largest recorded point source outbreak of human cryptosporidiosis in Sub-Saharan Africa and the only such outbreak to affect British military personnel anywhere. The recreational water sources were implicated by exposure histories taken from those affected, supported by environmental observations of visible contamination of the implicated sources, where gross fecal coliform contamination of the water was confirmed by testing. Conversely, nonimplicated water sources were free of coliforms. A single novel family subtype of *Cryptosporidium hominis* was found in almost all cases, supporting a common source of outbreak infection and expanding the geographical as well as host range of this particular subtype, which had previously been seen only in primates in China.

Early etiological identification of diarrhea in remote environments is often limited or delayed by access to diagnostics, affecting capacity for clinical decision-making and control measures. We have confirmed the feasibility and value of on-site multiplex molecular methods to detect enteropathogens in such a setting [6]. Further, we demonstrate its effective use in an unusual outbreak of cryptosporidiosis in military personnel, supporting evidence of suspected water point source, followed by person-to-person transmission. During the first few weeks of this outbreak, a single subtype of *C. hominis* predominated, as shown in the pathogen distribution timelines in Table 2. As infections with that subtype subsequently declined, a variety of bacterial and viral causes of gastroenteritis were identified, as observed during previous exercises in this setting [15]. This is in line with typical exposures, for example, the opportunity to eat at local community establishments in week 7 onwards for personnel returning from field conditions. Likewise, 4 non-*Cryptosporidium* cases during the same week demonstrated an epidemiologically linked *Campylobacter* spp. infection following a survival exercise and consumption of self-prepared poultry in field conditions by a small group of soldiers. Our experience highlights the importance of molecular diagnostics in informing infection control and antimicrobial treatment decisions, while also contributing to broader antibiotic stewardship efforts.

*Cryptosporidium* is known to circulate in both human and domesticated animal hosts in Kenya [28], with 9.8% prevalence reported in human fecal samples in nearby Nakuru County [29]. More than 97% of positive samples were identified during the rains, implicating surface runoff water or contaminated soil as a point source [29]. This outbreak occurred at the end of the dry season, and epidemiological investigations concluded that infection was associated with surface water exposure during recreational activities; environmental investigations showed raw sewage emptying into rivers utilized for such activities supported by fecal indicator test results. Domestic camp water contained no fecal contaminants, supporting our hypothesis of a point source outside camp. Detection of *Cryptosporidium* spp. in water is complicated by the typically low concentration of oocysts in water samples, with recovery relying on cartridge/membrane filtration or continuous-flow centrifugation of large volumes of water (minimum 10L) for analysis in specialist laboratories [30].

Molecular characterization of the *gp60* gene unexpectedly demonstrated infection with a novel highly divergent *C. hominis* ImA13G1 subtype. As far as we are aware, there is only one other report of subtype family Im, found in farmed crab-eating macaques in China infected with ImA13 [31]. *Cryptosporidium* spp. DNA was not confirmed in 10 stored samples that had previously tested positive on the FilmArray. There was no previous exposure history from the IeA11G3T3 subtype case whose first 3 samples did not amplify. This individual had been admitted to prolonged isolation following his first positive FilmArray result; however, the question of re-infection in the absence of supporting epidemiological evidence remains anecdotal. Our novel findings demonstrate human infection with a *C. hominis* subtype family across a far greater geographic spread than previously reported. Although it has been suggested that subtype family Im may cause a greater intensity of oocyst shedding than other subtypes in macaques [31], further studies are required to confirm this.

Contaminated recreational water is well documented as a source of cryptosporidiosis outbreaks in higher-income settings [32, 33]. Cryptosporidiosis outbreaks are rarely reported in Sub-Saharan Africa, partly due to a level of population immunity in endemic areas [34], but limitations in testing, reporting, recording, and investigation of cases must be considered.

Prolonged diarrhea symptoms and morbidity were observed in cryptosporidiosis cases in contrast to diarrheagenic *E. coli* cases, in keeping with experience elsewhere with cryptosporidiosis [2, 9, 35] and local experience with gastrointestinal infections in military personnel [15, 17]. Persistent diarrhea is the most commonly reported long-term sequela of cryptosporidiosis [35], which might explain ongoing diarrhea even among individuals with negative FilmArray results on retesting. Military public health decisions are largely influenced by Force Health Protection measures to minimize operational effectiveness risk and may therefore be more stringent than civilian responses. For example, as oocyst shedding is known to persist post–cessation of diarrhea, requiring a low infectious dose [36], asymptomatic military food handlers were removed from culinary duties until they provided negative fecal PCR samples, as opposed to civilian advice, which endorses return to duty >48 hours post–resolution of diarrhea [37].

There are several limitations to this study. The primary research objective before joining the deployment was to investigate different molecular diagnostic techniques for travelers’ diarrhea. Resources supplied for this were insufficient to conduct a full case–control study of exposure risks on arrival in Kenya just after this outbreak started. Recruitment of affected and asymptomatic personnel for a case–control study was also impractical in the circumstances; the population at risk was comprised of highly peripatetic groups located across a wide geographical range. Only probable and confirmed cases were isolated (at several locations) to curtail infection spread. The remaining nonexposed exercising troops were dispersed widely in different locations, and collection of detailed epidemiological data using formal questionnaires in field conditions was not possible. Although clinical data were adequately recorded and of good quality, environmental investigations were hampered by the logistics of conducting many detailed questionnaire-based interviews simultaneously over a wide area. These data were not recorded or stored in a standardized and retrievable manner; therefore, we are unable to present a systematic post hoc analysis. This highlights the importance of having adaptable interview templates and record storage methods, including the need for adequate resource and manpower allocation in future outbreaks, particularly in austere environments. The lack of capability to confirm potential water sources of infection, both in-country and with repatriated water samples, further complicated source attribution. However, the epidemiological links established during environmental investigations and clinical interviews all pointed to a common source.

Detection of *Cryptosporidium* spp. in environmental water is complicated by a low concentration of oocysts in water samples, with recovery relying on cartridge/membrane filtration or continuous-flow centrifugation of large volumes of water (minimum 10L) for analysis in specialist laboratories [30]. The FilmArray is not validated for testing water, and samples tested onsite using this platform were unsurprisingly negative for *Cryptosporidium* spp. The recovered six 150-mL water samples were stored and repatriated in an “off-label” manner for analysis, which might explain the inability to detect *Cryptosporidium* spp. on later testing.

The inability of the FilmArray to differentiate between live and dead enteropathogens was also a potential limitation. *Cryptosporidium* spp. may be present in feces for several weeks after resolution of clinical symptoms, complicating the distinction between ongoing infection and reinfection, as opposed to postinfection, shedding in individual cases. Although many personnel presenting with diarrhea were tested, universal testing was impractical. In outbreak scenarios in the United Kingdom, testing of samples from epidemiologically related cases with similar clinical features is often limited to a small number of index cases. Indeed, the military emphasis on maintaining operational effectiveness may outweigh the need for data collection in individuals who at this point could have become asymptomatic. As a result, the analysis of clinical syndromes across groups attributed to different pathogens was limited.

## CONCLUSIONS

As far as we are aware, this is the first published diarrheal outbreak in tropical military settings in which the FilmArray has been used for real-time testing. The utility of multiplex PCR has been shown to directly benefit case management, decision-making in remote settings, and instigation of outbreak control measures. It has enabled the identification and management of the largest outbreak of cryptosporidiosis described in Sub-Saharan Africa, and the first such large outbreak affecting British forces personnel anywhere. Our novel findings of primate-related *C. hominis* subtype family Im as the main cause suggest a potential expansion of the host range of this subtype from primates to humans, and then between humans, with a larger geographical range than previously recognized. Evidence on the intensity of oocyst shedding by the *C. hominis* ImA13G1 subtype is scant, and further studies investigating possible differences in clinical phenotypes are required. A greater understanding of the distribution and hosts of locally circulating *C. hominis* subtypes and their characterization is an important area for further research. This is vital for understanding the growing potential for zoonotic infection and for effective risk management to minimize the burden and impact of future cryptosporidiosis outbreaks.

## Supplementary Material

ofae001_Supplementary_Data

## Supplementary Data

Supplementary materials are available at *Open Forum Infectious Diseases* online. Consisting of data provided by the authors to benefit the reader, the posted materials are not copyedited and are the sole responsibility of the authors, so questions or comments should be addressed to the corresponding author.
