# Supplementary material for: Outbreak of Diarrhea Caused by a Novel Cryptosporidium hominis Subtype During British Military Training in Kenya
Source: Open Forum Infect Dis. 2024 Jan 3;11(1):ofae001. doi: 10.1093/ofid/ofae001 (PMC10798851; doi:10.1093/ofid/ofae001)
Supplement: ofae001_Supplementary_Data [file ofae001_supplementary_data.zip › 20231208-OFID_Supplementary Table 3.docx]

**Supplementary Table 3. Water testing results:** The results below show a mixture of some of the different sites tested with both pass and fail results for faecal coliforms and *Escherichia coli* (*E. coli*). Water testing was performed on-site with Colilert water testing kits (IDEXX, UK). Faecal coliform levels >1 colony forming units (cfu)/100ml indicate contamination. *E. coli* levels should be < 1 cfu/100ml. Contemporaneous Environmental Health (EH) notes from the investigating are included. The names of rivers and respective adventure training (AT) sites have been redacted and the point source results are annotated in red. All locations are external to the main operating base, but of note that water at Locations D and E is from the same source as the water used in the main base. The other locations use local or river water for both domestic purposes including swimming, and early epidemiological investigations showed a link between cryptosporidiosis cases and recent visit to Location C. There are no results from Location F, another adventure training site.

**Location A. River water source samples -** EH notes: Serials 1 and 2 are from two water sources that are normally use by the engineers to take water from and treat, in order to create potable water. This year both locations were not used. River located xx km south of Nyati Barracks. Not used due to a lack of quantity.

| Serial | Location/Equipment | Pass/Fail | Faecal coliforms  (cfu/100ml | *E. coli* cfu/100ml |
| --- | --- | --- | --- | --- |
| 1 | Location A. filtered by Seon UV | FAIL | 4 | Nil |
| 2 | Location A. Slow flow sample | FAIL | 344 | 52 |
| 3 | Location A. upstream fast flow | PASS | - | - |
| 4 | Location A. fast flowing | FAIL | 193.6 | 55 |

**Location B. River water source samples -** EH notes: Located xx km north of Nyati Barracks. These numbers may be elevated due to severe drought and extremely low water levels within the lake which may have meant the samples were extremely concentrated. However, I do not have base figures from the previous years to compare with. But it is my belief that the levels were concentrated. This water source was out of bounds.

| Serial | Location/Equipment | Pass/Fail | Faecal coliforms  (cfu/100ml | *E. coli* cfu/100ml |
| --- | --- | --- | --- | --- |
| 1 | Seon UV | FAIL | More than 2419.6 | 99 |
| 2 | C1 | FAIL | More than 2419.6 | 99 |
| 3 | Location B. | FAIL | More than 2419.6 | 99 |

**Location C. Adventure training site water source samples -** EH notes: As you are aware this was the AT site that was at the centre of our epidemiological data. I believe this location to be the epicentre for all cases, following exposure to the water course during AT activity. Again, severe drought may have a small role to play here in concentration levels. However, the river was still fast flowing so less likely to be of any serious concern. The fact that we are getting these levels within a river that is fast flowing is alarming.

This location has two swimming pools both of which do not have a working filtration system, nor do they have any pumps that agitate the water. The staff had zero understanding of the chlorination process and were seen just to throw unquantifiable levels of chlorine powder into the pool. On the day of sampling both swimming pools at this site appeared to have just been treated. This was apparent because a very large mound of undissolved chlorine was visible on the pool floor in one of the pools. Both pools are filled with river water and one of them failed. Chlorine does not kill cryptosporidium!

| Serial | Location | Pass/Fail | Faecal coliforms  (cfu/100ml | *E. coli* cfu/100ml | Action  Required |
| --- | --- | --- | --- | --- | --- |
| 1 | Location C. Drinking Water | PASS | - | - | - |
| 2 | Location C. River Water 2km Upstream | FAIL | More than 2419.6 | 98 | - |
| 3 | Location C. 2Km Downstream | FAIL | More than 2419.6 | 98 | - |
| 4 | Location C. swimming pool close to reception | FAIL | 4 | Nil | Retest after swimming pool chlorination.  Do not use until a retest is conducted by EHP |
| 5 | Location C. swimming pool B | PASS | - | - | - |

**Location D. Water source samples –** Location D is a Forward Operating Base and Location E is the Main Operating Base. The samples are from the water storage points used for drinking, bathing and cooking and this water originates from Nyati where it is treated, tested and shipped out. These two locations are furthest away from Nyati barracks, some 4 hours in either direction. The samples taken and the results received demonstrate the integrity of the water process within Nyati, ran by xx.

| Serial | Location | Pass/Fail | Faecal coliforms  (cfu/100ml | *E. coli* cfu/100ml |
| --- | --- | --- | --- | --- |
| 1 | Location D. Ablution | PASS | - | - |
| 2 | Location D. Showers | PASS | - | - |
| 3 | Location D. treatment plant | PASS | - | - |
| 4 | Location D. Kitchen | PASS | - | - |
| 5 | Location D. Laundry | PASS | - | - |

**Location E. Water source samples**

| Serial | Location | Pass/Fail | Faecal coliforms  (cfu/100ml | *E. coli* cfu/100ml |
| --- | --- | --- | --- | --- |
| 1 | Location E. Shower | PASS | - | - |
| 2 | Location E. treatment plant | PASS | - | - |
| 3 | Location E. Kitchen | PASS | - | - |
| 4 | Location E. Laundry | PASS | - | - |
| 5 | Location E. Ablution | PASS | - | - |
