# Supplementary material for: Outbreak of Diarrhea Caused by a Novel Cryptosporidium hominis Subtype During British Military Training in Kenya
Source: Open Forum Infect Dis. 2024 Jan 3;11(1):ofae001. doi: 10.1093/ofid/ofae001 (PMC10798851; doi:10.1093/ofid/ofae001)
Supplement: ofae001_Supplementary_Data [file ofae001_supplementary_data.zip › 20231208-OFID_Supplementary Table 1.docx]

**Supplementary Table 1.** **PCR BioFire^®^ FilmArray^®^ GI Panel targets**

The 22 enteropathogens targeted by the PCR BioFire® FilmArray® directly from faecal specimens, after Buss SN *et al* [E]

| **Bacteria** | **Diarrhoeagenic *E. coli or Shigella spp.*** |
| --- | --- |
| - *Campylobacter* (*jejuni, coli* & *upsaliensis*) - *Clostridium difficile* (Toxin A/B) - *Plesiomonas* *shigelloides* - *Salmonella spp.* - *Yersinia enterocolitica* - *Vibrio* (*parahaemolyticus, vulnificus*, & *cholerae*) - *Vibrio cholerae* | - *E. coli* O157 - Enteroaggregative *E. coli* (EAEC) - Enteroinvasive *E. coli* (EIEC) - Enteropathogenic *E.* coli (EPEC) - Enterotoxigenic *E. coli* (ETEC) lt/st - Shiga-like toxin-producing *E. coli* (STEC) stx1/stx2 - *E. coli* O157 - *Shigella*/Enteroinvasive *E. coli* (EIEC) |
| **Viruses** | **Parasites** |
| - adenovirus F 40/41 - astrovirus - norovirus GI/GII - rotavirus A - sapovirus (I, II, IV, and V) | - *Cryptosporidium* spp. - *Cyclospora cayetanensis* - *Entamoeba histolytica* - *Giardia* *duodenalis** |

* Also known as *Giardia lamblia* or *Giardia intestinalis*
