# Supplementary material for: Outbreak of Diarrhea Caused by a Novel Cryptosporidium hominis Subtype During British Military Training in Kenya
Source: Open Forum Infect Dis. 2024 Jan 3;11(1):ofae001. doi: 10.1093/ofid/ofae001 (PMC10798851; doi:10.1093/ofid/ofae001)
Supplement: ofae001_Supplementary_Data [file ofae001_supplementary_data.zip › 20231208-OFID_Supplementary Table 2.docx]

**Supplementary Table 2. Clinical characteristics of 51 individuals presenting with diarrhoea and confirmed PCR results via BioFire^®^ FilmArray^®^.**

Mixed PCR results consisted of: *Cryptosporidium* spp. with EPEC^1^ (7), ETEC^2^ (3), *Salmonella* spp. (1), *Campylobacte*r spp. (1), EPEC and *Clostridioides difficile* (1), astrovirus (1), EAEC^3^, EPEC, ETEC (1) and EPEC, EAEC with astrovirus (1). Other pathogen only results consisted of: *Campylobacter* spp. (3), *Campylobacte*r spp. and EPEC (1), STEC^4^ (1), EPEC (3), EAEC (1). Individuals with *Cryptosporidium* results (Group 1 and 2) compared with individuals that had positive PCR results for those with only non-*Cryptosporidium* pathogens (Group 3). Comparisons made by one-way ANOVA or Chi squared test (vomiting and abdominal cramps).

IQR - Interquartile range,

SD - Standard deviation

^1^EPEC - Enteropathogenic *Escherichia coli*

^2^ETEC - Enterotoxigenic *Escherichia coli*

^3^EAEC - Enteroaggregative *Escherichia coli*

^4^STEC - Shiga toxin-producing *Escherichia coli*

| **PCR result** | **Group 1**  *Cryptosporidium* spp. only  *(n=26)* | **Group 2**  *Cryptosporidium* spp. with ≥ 1 other pathogen *(n=16)* | **Group 3**  Other pathogens only  *(n=9)* | **Groups 1 and 2 compared to group 3**  (*p* value) |
| --- | --- | --- | --- | --- |
| **Age, median (IQR) years** | 25 (7) | 24 (10) | 25 (16.5) | 0.12 |
| **Duration of diarrhoea, mean (SD) days** | 7.7 (5.0) | 7.6 (3.9) | 2.3 (0.9) | 0.001 |
| **Days in isolation, mean (SD) days** | 8.9 (5.7) | 7.1 (4.1) | 3.3 (1.1) | 0.002 |
| **Number of stools per day, mean (SD)** | 4.4 (1.9) | 3.9 (2.7) | 3.7 (1.49) | 0.65 |
| **Peak temperature recorded, mean (SD) ^o^C** | 37.1 (0.38) | 37.2 (0.63) | 37.4 (0.69) | 0.30 |
| **Vomiting**  ***n* (%)** | 5 (19.2%) | 1 (6.3%) | 3 (33.4%) | 0.17 |
| **Abdominal cramps**  ***n* (%)** | 13 (50%) | 9 (56.3%) | 4 (44.4%) | 0.67 |
